# Psychomotor Symptoms in Chronic Cocaine Users: An Interpretative Model

**DOI:** 10.3390/ijerph19031897

**Published:** 2022-02-08

**Authors:** Davide Cenci, Manuel Glauco Carbone, Camilla Callegari, Icro Maremmani

**Affiliations:** 1 Department of Translational Medicine, Major University Hospital of Charity, Psychiatry Institute, Eastern Piedmont University, Via Solaroli 17, 28100 Novara, Italy; davide.cenci89@gmail.com; 2 Department of Medicine and Surgery, Division of Psychiatry, University of Insubria, Viale Luigi Borri 57, 21100 Varese, Italy; manuelglaucocarbone@gmail.com (M.G.C.); camilla.callegari@uninsubria.it (C.C.); 3 Pisa-School of Experimental and Clinical Psychiatry, Via di Pratale 2, 56100 Pisa, Italy; 4 VP Dole Dual Disorder Unit, G. Delisio Institute of Behavioral Sciences, Via di Pratale 2, 56100 Pisa, Italy; 5 Saint Camillus International University of Health and Medical Sciences, Via di Sant’Alessandro 8, 00131 Rome, Italy

**Keywords:** cocaine use disorder, extrapyramidal symptoms, long-term cocaine abuse, schizoaffective disorder, antipsychotic-induced side effects

## Abstract

According to the latest estimates, there are around 24.6 million cocaine users worldwide, and it is estimated that around a quarter of the population worldwide has used cocaine at some point in their lifetime. It follows that such widespread consumption represents a major risk for public health. Long-term use of cocaine, in addition to being related to many cerebral and cardiovascular diseases, is increasingly associated with a higher incidence of psychomotor symptoms and neurodegenerative disorders. In recent years, numerous studies have shown an increased risk of antipsychotic-induced extrapyramidal symptoms (EPSs) in patients with psychotic spectrum disorders comorbid with psychostimulant misuse, particularly of cocaine. In the present paper, we describe the case of a young patient on his first entry into a psychiatric setting with previous cocaine misuse who rapidly presented psychomotor symptoms and was poorly responsive to symptomatic therapy consisting of benzodiazepines and anticholinergics, in relation to the introduction of various antipsychotics (first, second, and third generation). Furthermore, we propose neurobiological mechanisms underlying the hypothesized increased vulnerability to psychomotor symptoms in chronic cocaine abusers. Specifically, we supposed that the chronic administration of cocaine produces important neurobiological changes, causing a complex dysregulation of various neurotransmitter systems, mainly affecting subcortical structures and the dopaminergic and glutamatergic pathways. We believe that a better understanding of these neurochemical and neurobiological processes could have useful clinical and therapeutic implications by providing important indications to increase the risk–benefit ratio in pharmacological choice in patients with psychotic spectrum disorders comorbid with a substance use disorder.

## 1. Introduction

The dopaminergic system plays important roles in neuromodulation, such as movement and motor control, spatial memory function, reinforcement, motivation, reward, attention, arousal, sleep regulation, affect, cognitive function, feeding, olfaction, lactation, and maternal and reproductive behaviors [1,2]. It also regulates the hormone balance and influences the immune, cardiovascular, gastrointestinal, and renal systems [3]. 

Dopamine (DA) is a neurotransmitter, synthesized in both the central nervous system and the periphery, that exerts its actions upon binding to G protein-coupled receptors [4]. DA receptors are widely expressed in the body and function in both the peripheral and the central nervous systems [5]. Dopaminergic signaling pathways are crucial to the maintenance of physiological processes, and an unbalanced activity may evoke the onset and progression of some diseases in the nervous system, such as schizophrenia (SZ), substance use disorder (SUD), Parkinson disease (PD), Huntington’s disease (HD), restless leg syndrome (RLS), and attention deficit and hyperactivity disorder (ADHD) [6]. Each pathway is a set of projection neurons, consisting of individual DA neurons. There are four major dopaminergic pathways: the *mesolimbic*, the *mesocortical*, the *nigrostriatal*, and the *tuberoinfundibular* pathways. Other dopaminergic pathways include the *hypothalamospinal* tract and the *incertohypothalamic* pathway [7]. 

Although inevitably a gross oversimplification, dopaminergic dysregulation in the mesocortical targets is involved in the symptomatology of SZ, while altered dopaminergic function in the mesolimbic pathway is pivotally implicated in the pathogenesis and neuropsychopharmacology of addiction. On the other hand, the work of the nigrostriatal pathway can, in broad terms, be thought of as the planning, initiation, and expression of movement [1]. 

Albeit the neurobiology of SZ and, in general, of psychotic spectrum disorders remains to be elucidated, dopaminergic dysregulation in the *mesocortical targets* has been proposed as one of the most accepted theories involved in the symptomatology of these diseases (“DA hypothesis”) [8]. The DA hypothesis of SZ postulates that the hyperactivity of DA receptor D_2_ neurotransmission in subcortical and limbic brain regions contributes to positive symptoms, whereas negative and cognitive symptoms of the disorder can be attributed to the hypofunctionality of DA receptor D_1_ neurotransmission in the prefrontal cortex [9]. The fact that DA-releasing drugs, such as amphetamine and cocaine, possess psychotomimetic properties, in addition to the D_2_ antagonist property common to many of the currently prescribed antipsychotic treatments, gives credence to the DA hypothesis of SZ [10,11]. 

At the same time, strong evidence indicates that synaptic alterations in *mesolimbic pathways* are related to drug and food addiction, and mutual neural substrates for both DA-dependent disorders have been described [12,13]. Specifically, it has been demonstrated that DA is involved in reward-related incentive learning [14,15]. A reward system is a group of neural structures responsible for incentive salience (i.e., “wanting”; desire or craving for a reward and motivation), associative learning (primarily positive reinforcement and classical conditioning), and positively valenced emotions, particularly those involving pleasure as a core component. A reward is the attractive and motivational property of a stimulus that induces appetitive and consummatory behaviors [16,17]. Studies in rats with chronic exposure to drugs of abuse, such as cocaine, showed adaptations of the dopaminergic system (VTA/NAc) caused by the upregulation of the DA receptor D_1_ and the downregulation of the DA receptor D_2_ [18].

The *nigrostriatal pathway* is the source of planning, initiation, and expression of movement. It is a bilateral dopaminergic pathway in the brain that originates in the substantia nigra pars compacta (SNc) and sends its projections to the caudate and putamen nuclei of the dorsal striatum [3]. Dopaminergic neurons of this pathway release DA from axon terminals that synapse onto GABAergic medium spiny neurons (MSNs), also known as spiny projection neurons (SPNs), located in the dorsal striatum [19].

Degeneration of dopaminergic neurons in the SNc is one of the main pathological features of PD, leading to a marked reduction in DA function and the symptomatic motor deficits of parkinsonism including hypokinesia, tremors, rigidity, and postural imbalance [20].

Treatments with antipsychotics, which are especially effective on the positive symptoms of psychotic spectrum disorders, were strongly related to the presence of extrapyramidal symptoms (EPSs) such as akathisia (subjective and observed restlessness), dystonia (muscular cramps), tardive dyskinesia (repetitive and involuntary movements), and parkinsonism (resting tremor, bradykinesia, and rigidity) [21]. The basic mechanisms underlying the emergence of these symptoms remain to be elucidated, but there is reliable evidence from positron emission tomography (PET) or single-photon emission computed tomography (SPECT) studies showing that EPSs emerge mainly when DA receptor D_2_ occupancy by antipsychotics is greater than approximately 80%, which was also associated with worse subjective experience and increased substance abuse [21]. 

Moreover, the problem of antipsychotic-induced EPSs is further compounded by the fact that psychotic spectrum disorders are associated with a nearly 50% lifetime prevalence of substance use disorders (SUDs) [22]. An aggravation of EPSs via administration of antipsychotics and/or some psychoactive substances such as alcohol, cocaine, amphetamine, and cannabis is conceivable, because these compounds have effects on the basal ganglia which are vital for voluntary movement [23]. In particular, cocaine and amphetamine stimulate nigrostriatal dopaminergic neurotransmission by blocking and reversing the DA transporter (DAT), respectively [24,25]. Chronic cocaine abusers have been found to have decreased levels of DA in the caudate nucleus and frontal cortex that are not associated with an increase in DA receptor gene expression [26]. Furthermore, long-term cocaine use is associated with a significant reduction in DA receptor D_2_ availability in the dorsal striatum that may last for months after withdrawal, similar to the striatal dopaminergic deficit observed in PD [27,28,29]. Nonpsychotic individuals with cocaine use disorder (CoUD) showed EPSs, including rigidity and parkinsonian resting tremor, up to 12 weeks after having discontinued use of the substance [23,25,30,31,32]. In patients with a diagnosis of SZ, cocaine use is a major risk factor for dystonia, parkinsonism, dyskinesia, and akathisia [23,33,34].

Consequently, the abuse of psychostimulants acting mainly on the dopaminergic pathways has a high impact both on positive symptoms and on motor symptoms related, or not, to the use of antipsychotics.

In the present paper, we describe the case of a young drug-naïve patient presenting psychotic symptoms with a history of CoUD who showed a marked tendency to the onset of antipsychotic-induced EPSs, partially responsive to anticholinergic drugs. At the same time, we propose the possible neurobiological processes underlying the long-term effects of cocaine on dopaminergic pathways in the predisposition of EPSs and motor symptoms.

## 2. Case Description

Mr. Z is a 23-year-old male patient, with a negative psychiatric history, who arrived at our emergency room reporting auditory misperceptions and megalomanic and persecutory ideas.

The patient, a healthy carrier of beta-thalassemia, did not suffer from any other current or past medical conditions and had no family history of psychiatric disorders. He referred to previous cocaine abuse and sporadic cannabis use.

At admission, on psychic examination, the patient presented logorrheic speech with a hyper-phonic tone. He was euphoric with mild motor restlessness and with a reduced need for sleep. He displayed slight acceleration of thoughts with structured megalomanic, paranoid, and persecutory ideas. He denied recent substance use.

He described auditory hallucinations and interpretative ideas linked to the suspicion of being kept under control, which started a few months earlier at work (he was a “rider” for delivery services). Since then, he suffered from a strong state of anguish, fear, hyperarousal, and persistent insomnia. The conviction of being controlled progressively intensified, first through his mobile phone and then through a direct connection to his nervous system. This brain network would have brought him to the knowledge of various secrets and conspiracies, also allowing him to interact with prominent personalities of the political and financial world scene. At first, he felt gratified for having been “chosen” for this role, but then he began to feel overwhelmed by the flow of information, seeking medical help to break the connections described.

Mr. Z reported a small but constant use of cocaine over time, from the age of 18, which he increased during the last year and then suspended about two months before his admission at our emergency room. The neurological examination, routine blood tests, and toxicological urine test (cocaine, opiates, cannabinoids, amphetamines, and ecstasy) were normal and confirmed no recent substance use. The patient accepted to cooperate with the proposed treatments, and he was admitted to the psychiatry ward. Aripiprazole 20 mg/day, valproic acid 500 mg/day, and delorazepam 3 mg/day were introduced.

From the first days, he appeared reassured by staying in a protected environment, and the circadian rhythm quickly recovered. After the second administration of aripiprazole, the patient began to present hiccups and dyspepsia that persisted for almost 24 h despite using chlorpromazine 25 mg. Aripiprazole was discontinued, and the hiccups gradually disappeared.

At the same time, paliperidone 6 mg/day (increased to 9 mg after two days) was introduced with a gradual improvement in the positive symptoms and the affective state. After seven days of hospitalization, the patient appeared calm and behaviorally adequate, aware of the need for care and prosecution of pharmacological treatment. The delusional ideation persisted but attenuated, with less emotional participation. He was discharged with the indication to continue pharmacological treatment under psychiatric control.

A few days after discharge, during the outpatient visit, Mr. Z described painful episodic involuntary muscle contractions in the back and neck and showed a psychomotor slowdown. Biperiden 4 mg/day was introduced, and paliperidone was reduced to 6 mg/day; however, there was no clinical improvement in the following days.

Consequently, paliperidone was discontinued, haloperidol 3 mg/day was introduced, and valproic acid was increased to 1000 mg/day. Additionally, in this case, within a few days, the patient developed an aggravating akathisia which led him to go to the emergency room, where he was administered intravenous diazepam and biperiden, with an improvement in symptoms (see Table 1). At the same time, the patient was recommended to be admitted to the psychiatric ward for a therapeutic re-evaluation (sixteen days after discharge). At admission, he showed restlessness, agitation, and inner tension with the urge to move constantly. The psychotic symptomatology was still active but nuanced and experienced with little emotional participation. He was euthymic, with the regular hypnic pattern. Blood and toxicological urine tests were normal.

A therapeutic switch was set from haloperidol to lurasidone 74 mg/day, and a progressive improvement in the psychomotor symptomatology was observed; after seven days, Mr. Z was finally discharged. In the following three months, during the check-ups, he no longer showed EPSs and had a further progressive reduction in psychotic symptoms with the recovery of social and work functioning.

## 3. Discussion

Cocaine use represents a significant problem worldwide, affecting millions of people and reaching an all-time high in terms of consumption in 2019 [35].

Possible ways to categorize the effects of cocaine are based on time characteristics, i.e., neurologic complications with acute or chronic use, or whether the patient is an active user, or early or late abstinent.

As previously mentioned, acute administration of cocaine induces a major rise in extracellular DA levels, blocking DAT and preventing the reuptake of DA, throughout the dorsal striatum (caudate nucleus and putamen) and prefrontal cortex in both humans and experimental animals. Furthermore, cocaine also increases the levels of other monoamines by blocking serotonin (5-HT) or norepinephrine (NE) transporters. Increased monoamine levels, specially DA, are thought to be involved in the euphoric effects of cocaine as well as explaining its motoric side effects [36].

In chronic users, in response to the elevated DA levels, DAT downregulation might take place, as a compensatory mechanism. This compensatory mechanism progressively attenuates the acute DA elevation related to cocaine intake, but in the long term, it leads to DA deficiency in the dorsal striatum and frontal cortex as reuptake is needed for synaptic storage and synthesis of DA [37]. Furthermore, as demonstrated by neuropathological studies, chronic use of cocaine implicates an overstimulation of the dopaminergic terminals and an excessive metabolism of the neurotransmitter. The “stress” to which the dopaminergic pathways are subjected progressively culminates in a loss of their efficiency, resulting in a reduced synthesis and release of DA [38]. Long-term psychostimulant use can increase lipid peroxidation and DNA oxidation as well as elevating levels of reactive oxygen species (ROS) such as hydroxyl and hydrogen peroxide. Oxidative stress to lipids and DNA of the cells within the target pathways results in neurotoxicity, specifically selective striatal nerve terminal damage and loss of DA in the NAc [39,40,41,42,43].

Dopaminergic terminals also have marked reductions in the vesicular monoamine transporter-2 (VMAT-2) [44]. Within presynaptic terminals of monoaminergic neurons, VMAT-2 translocates monoamines (DA, 5-HT, NE, and histamine) from the cytosol across the vesicle membrane into the vesicle lumen following neurotransmitter biosynthesis and/or clearance from the extracellular space [45]. In line with these findings, in post-mortem and in vivo human studies, cocaine use has been found to diminish striatal and mesencephalic DA neuronal markers, which suggests that in humans, cocaine use causes a loss of DA neurons, predisposing individuals to neurodegenerative diseases such as PD [46]. On the other hand, depletion of DA levels is not paralleled by an increase in D_1_ and D_2_ receptor gene expression.

Moreover, recent studies showed that chronic cocaine exposure results in an abnormal increase in spine density (spinogenesis) on MSNs, introducing another level of cocaine-induced morphological plasticity [47,48,49]. It has been hypothesized that these morphological changes influence mesolimbic, mesocortical, and nigrostriatal pathways and may contribute to the development of reduced behavioral sensitivity, behavioral sensitization, and compulsive patterns of drug-seeking behaviors as well as psychomotor and positive symptoms [50,51]. In particular, it also appears that the D_1_-positive “direct pathway” neurons are more sensitive to cocaine-induced changes in spine density than D_2_-containing “indirect pathway” neurons [52,53]. Calcium-mediated regulation of myocyte enhancer factor 2 (MEF2) may contribute to the changes in spine density. Although MEF2 proteins are widely expressed in the CNS, their role has long remained enigmatic until recent studies showing that MEF2 regulates excitatory synapses, in part, by promoting activity-dependent synaptic pruning [32,54,55]. A scheme by which chronic cocaine exposure increases dendritic spine density via a reduction in MEF2-dependent transcription was recently suggested. The abnormal increase in DA and the resulting overactivation of the DA receptor D_1_ and its cAMP-dependent signaling cascade, combined with the delta FosB-mediated increases in cyclin-dependent kinase 5 (Cdk5), result in a significant attenuation of MEF2 activity and increased spine density in D_1_-containing NAc neurons [51].

**Figure 1:**
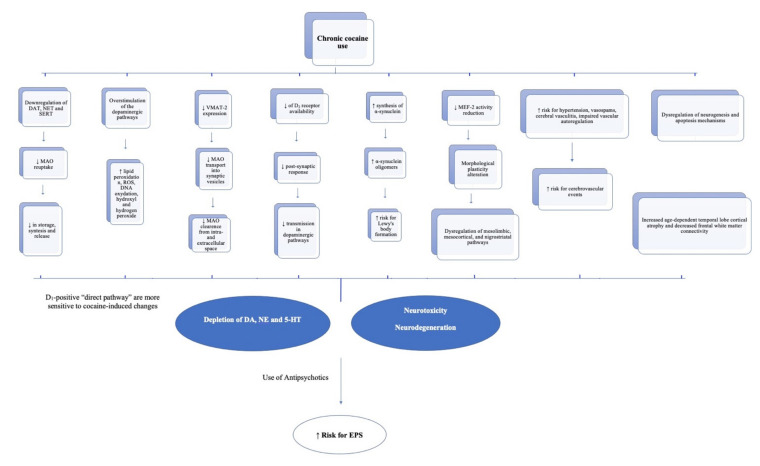
Neurobiological changes induced by chronic cocaine use.

It should be mentioned that the above-described mechanisms are speculative, and the effects of cocaine on the nervous system are complex. We also need to consider changes in D_2_ receptor expression and possible long-term structural damage to dopaminergic synaptic terminals [56]. Long-term cocaine abuse is associated with a significant reduction in DA receptor D_2_ availability in the dorsal striatum that may last for months after detoxification, similar to the striatal dopaminergic deficit observed in PD (Figure 1) [25].

Considering parkinsonism, it is rarely described as a result of cocaine use, and, in fact, inhaled cocaine has been reported to ameliorate parkinsonian “off” periods in self-medicating patients without triggering dyskinesias [57]. However, in the neurobiological processes described above, chronic cocaine use can cause parkinsonian features that may persist after drug withdrawal. In line with these considerations, overexpressed alpha-synuclein has been observed in midbrain dopaminergic neurons from cocaine abusers, possibly representing a neuroadaptive response to the exposure and potentially exposing them to the risk of degenerative changes in dopaminergic neurons [58]. Aggregations of the protein alpha-synuclein compose an intracellular inclusion called the “Lewy body” that represents the pathological hallmark of PD [59]. Moreover, individuals with a history of primarily psychostimulant use exhibit an abnormal SN morphology. Using transcranial sonography, hyperechogenicity of SN was detected, and it was long lasting and did not appear to be associated with concurrent cannabis use. The hyperechogenicity observed in stimulant users was comparable to older adults with clinical PD. Increased activation of microglia was associated with SN nigra hyperechogenicity [60].

Finally, we should not forget that the medical and neurological complications of cocaine use are well known. Among the neurological complications, cocaine is known to cause cerebrovascular events (both ischemic and hemorrhagic) via diverse mechanisms including induced hypertension, vasospasm, cerebral vasculitis, and impaired vascular autoregulation [61]. Chronic cocaine abuse led to increased age-dependent temporal lobe cortical atrophy and decreased frontal white matter connectivity shown by imaging studies [62].

Consequently, cocaine misuse could be associated with EPSs, particularly in patients treated with antipsychotics.

In our case, patient Mr. Z had a history of cocaine abuse, which stopped a few months before admission but had previously lasted for years. *Hiccups* were the first iatrogenic adverse effect reported by the patient, and they are a known side effect of aripiprazole [63]. While the reason for this effect is not completely clear, it is thought to be related to dopaminergic dysregulation and considered to be within the EPS group [64]. *Dystonia*, *akathisia*, and *parkinsonism* are known psychomotor symptoms related to central dopaminergic dysregulation [65], and in the case described, they appeared quickly, within a few days, causing a major impact on the quality of life. These EPSs were refractory to symptomatic treatments and regressed only after the suspension of the antipsychotics in use.

Although EPSs do not solely rely on striatal DA dysfunction, but rather represent a complex network of symptoms that affect multiple neurochemical entities, striatal DA remains a key underlying factor of the neurochemical and pathophysiological changes associated with these entities.

## 4. Proposal

The present paper described the case of a patient with psychotic symptoms, mood fluctuations (manic/hypomanic episodes), and a history of cocaine misuse, admitted to our psychiatric department, who was treated with different antipsychotics and had developed many EPSs. Based on the longitudinal evaluation, a diagnosis of schizoaffective disorder could be made according to the diagnostic criteria of DSM 5 [66].

The case described seems to confirm that long-term cocaine misuse, due to its complex effects on the central nervous system, confers a greater vulnerability to the onset of psychomotor symptoms to patients. Mr. Z showed different EPSs despite having already suspended the substance abuse for some months.

As described above, chronic administration of cocaine causes important neurobiological changes, particularly affecting the dopaminergic and glutamatergic pathways, which make patients particularly sensitive to antipsychotic-induced EPSs. This vulnerability is not related to the type of antipsychotics used (first, second, and third generation), and it is also evident at relatively low dosages and is partially responsive to the combination of anticholinergic drugs and benzodiazepines.

On the other hand, we cannot fully explain how Mr. Z did not present EPSs following having taken lurasidone. The high antagonist activity of lurasidone on the 5-HT receptor 5-HT_2A_ is believed to justify the low incidence of EPSs compared to haloperidol and aripiprazole, but not to paliperidone. The resulting decreased activity in the nigrostriatal and mesolimbic serotonergic pathways increases dopaminergic transmission, opposing the D_2_ antagonist activity which, in association with the intake of biperiden, limits the onset of EPSs, even in a more vulnerable dopaminergic system such as that of Mr. Z.

This suggests a double interpretation: patients being treated with antipsychotic drugs should refrain from using cocaine, in order not to increase the risk of running into adverse reactions; at the same time, being aware of a patient’s history of previous cocaine abuse should guide the physician towards a therapeutic approach that reduces the risk of onset of EPSs as much as possible, to which these patients are likely to have a greater susceptibility. Furthermore, it is possible to hypothesize that the dopaminergic dysregulation related to the chronic use of cocaine might trigger, unmask, and worsen psychotic symptoms and might lower the responsiveness to antipsychotic treatments. Finally, antipsychotics worsen the severity of the SUD by increasing “craving” and reducing “reward”, further worsening the clinical symptomatology.

The choice of the most appropriate treatment leads to a compromise that allows for good management of the psychiatric symptomatology and the eventual onset of side effects, even with the addition of a specific symptomatic therapy.

Adverse effects can therefore also significantly affect mental well-being, as well as making it difficult to set up an effective treatment, forcing multiple changes in pharmacological therapy with results that are not always optimal. Knowing any history of cocaine abuse in patients whose symptoms require the use of antipsychotics can immediately lead to the exclusion of drugs that present a greater risk of causing EPSs, which may prove unsuitable.

To make an even more accurate choice of treatment of psychotic symptoms in patients with SUD possible, it would be useful to perform further studies analyzing the medium- and long-term impacts of specific substances on the CNS, any interactions with antipsychotics, and the efficacy of other pharmacological associations (for example, focusing more on mood stabilizers), as well as looking for any other reasons for interindividual variability in the development of adverse reactions to antipsychotic drugs.

## 5. Conclusions

In conclusion, we suppose that the chronic administration of cocaine produces important neurobiological changes, causing a complex dysregulation of various neurotransmitter systems, mainly affecting subcortical structures and the dopaminergic and glutamatergic pathways.

## Figures and Tables

**Table 1:** Pharmacological therapy, side effects, treatments, and outcomes.

Drugs	Side Effects	Treatments	Outcomes
Aripiprazole 20 mg/day After the second administration of 10 mg	Protracted hiccups and dyspepsia for 24 h	Chlorpromazine 25 mg and aripiprazole suspension	Gradual resolution in 2–3 h
Paliperidone 9 mg/day About 8–10 days after the introduction	Painful dystonia and muscle contractions in the back and neck	Introduction of biperiden and reduction in paliperidone to 6 mg/day	Poor response ↓ paliperidone suspension
Haloperidol 3 mg/day About 4–6 days after the introduction	Aggravating akathisia and psychomotor agitation	Biperiden 5 mg IV and diazepam 10 mg IV	Complete resolution

## References

[1] Klein M.O., Battagello D.S., Cardoso A.R., Hauser D.N., Bittencourt J.C., Correa R.G. (2019). Dopamine: Functions, Signaling, and Association with Neurological Diseases. Cell. Mol. Neurobiol..

[2] Baik J.-H. (2020). Stress and the dopaminergic reward system. Exp. Mol. Med..

[3] Alcaro A., Huber R., Panksepp J. (2007). Behavioral functions of the mesolimbic dopaminergic system: An affective neuroethological perspective. Brain Res. Rev..

[4] Beaulieu J.-M., Gainetdinov R.R. (2011). The Physiology, Signaling, and Pharmacology of Dopamine Receptors. Pharmacol. Rev..

[5] Baik J.-H. (2013). Dopamine Signaling in reward-related behaviors. Front. Neural Circuits.

[6] Kim E.K., Choi E.-J. (2010). Pathological roles of MAPK signaling pathways in human diseases. Biochim. Biophys. Acta BBA Mol. Basis Dis..

[7] Serafini R.A., Pryce K.D., Zachariou V. (2020). The Mesolimbic Dopamine System in Chronic Pain and Associated Affective Comorbidities. Biol. Psychiatry.

[8] Kambeitz J., Abi-Dargham A., Kapur S., Howes O. (2014). Alterations in cortical and extrastriatal subcortical dopamine function in schizophrenia: Systematic review and meta-analysis of imaging studies. Br. J. Psychiatry.

[9] Toda M., Abi-Dargham A. (2007). Dopamine hypothesis of schizophrenia: Making sense of it all. Curr. Psychiatry Rep..

[10] Fiorentini A., Volonteri L.S., Dragogna F., Rovera C., Maffini M., Mauri M.C., Altamura C.A. (2011). Substance-induced psychoses: A critical review of the literature. Curr. Drug Abus. Rev..

[11] Molitch M.E. (2020). Dopamine agonists and antipsychotics. Eur. J. Endocrinol..

[12] Kenny P.J. (2011). Common cellular and molecular mechanisms in obesity and drug addiction. Nat. Rev. Neurosci..

[13] Baik J.-H. (2013). Dopamine signaling in food addiction: Role of dopamine D2 receptors. BMB Rep..

[14] Pessiglione M., Seymour B., Flandin G., Dolan R.J., Frith C.D. (2006). Dopamine-dependent prediction errors underpin reward-seeking behaviour in humans. Nature.

[15] Pessiglione M., Schmidt L., Draganski B., Kalisch R., Lau H., Dolan R.J., Frith C.D. (2007). How the Brain Translates Money into Force: A Neuroimaging Study of Subliminal Motivation. Science.

[16] Schultz W. (2015). Neuronal Reward and Decision Signals: From Theories to Data. Physiol. Rev..

[17] Berridge K.C., Kringelbach M. (2015). Pleasure Systems in the Brain. Neuron.

[18] Lynch W.J., Peterson A.B., Sanchez V., Abel J., Smith M.A. (2013). Exercise as a novel treatment for drug addiction: A neurobiological and stage-dependent hypothesis. Neurosci. Biobehav. Rev..

[19] Tritsch N.X., Ding J.B., Sabatini B.L. (2012). Dopaminergic neurons inhibit striatal output through non-canonical release of GABA. Nature.

[20] Dorsey E.R., Sherer T., Okun M.S., Bloem B.R. (2018). The Emerging Evidence of the Parkinson Pandemic. J. Park. Dis..

[21] Waddington J.L. (2020). Psychosis in Parkinson’s disease and parkinsonism in antipsychotic-naive schizophrenia spectrum psychosis: Clinical, nosological and pathobiological challenges. Acta Pharmacol. Sin..

[22] Khokhar J.Y., Dwiel L.L., Henricks A.M., Doucette W.T., Green A.I. (2018). The link between schizophrenia and substance use disorder: A unifying hypothesis. Schizophr. Res..

[23] Potvin S., Pampoulova T., Mancini-Marië A., Lipp O., Bouchard R.-H., Stip E. (2006). Increased extrapyramidal symptoms in patients with schizophrenia and a comorbid substance use disorder. J. Neurol. Neurosurg. Psychiatry.

[24] Dela Peña I., Gevorkiana R., Shi W.-X. (2015). Psychostimulants affect dopamine transmission through both dopamine transporter-dependent and independent mechanisms. Eur. J. Pharmacol..

[25] Zhornitsky S., Stip E., Pampoulova T., Rizkallah E., Lipp O., Bentaleb L.A., Chiasson J.-P., Potvin S. (2010). Extrapyramidal symptoms in substance abusers with and without schizophrenia and in nonabusing patients with schizophrenia. Mov. Disord..

[26] Cadet J.L., Jayanthi S., McCoy M.T., Beauvais G., Cai N.S. (2010). Dopamine D1 receptors, regulation of gene expression in the brain, and neurodegeneration. CNS Neurol. Disord. Drug Targets.

[27] Trifilieff P., Martinez D. (2014). Imaging addiction: D2 receptors and dopamine signaling in the striatum as biomarkers for impulsivity. Neuropharmacology.

[28] Ashok A.H., Mizuno Y., Volkow N.D., Howes O.D. (2017). Association of Stimulant Use with Dopaminergic Alterations in Users of Cocaine, Amphetamine, or Methamphetamine: A Systematic Review and Meta-analysis. JAMA Psychiatry.

[29] Volkow N.D., Fowler J.S., Wang G.-J., Swanson J.M., Telang F. (2007). Dopamine in Drug Abuse and Addiction: Results of imaging studies and treatment implications. Arch. Neurol..

[30] Lappin J.M., Sara G.E. (2019). Psychostimulant use and the brain. Addiction.

[31] Satel S.L., Swann A.C. (1993). Extrapyramidal symptoms and cocaine abuse. Am. J. Psychiatry.

[32] Potvin S., Blanchet P., Stip E. (2009). Substance abuse is associated with increased extrapyramidal symptoms in schizophrenia: A meta-analysis. Schizophr. Res..

[33] Maat A., Fouwels A., De Haan L. (2008). Cocaine is a major risk factor for antipsychotic induced akathisia, parkinsonism and dyskinesia. Psychopharmacol. Bull..

[34] Green A.I. (2005). Schizophrenia and comorbid substance use disorder: Effects of antipsychotics. J. Clin. Psychiatry.

[35] United Nations Office on Drugs and Crime (UNODC) (2021). World Drug Report 2021.

[36] Drake L.R., Scott P.J.H. (2018). DARK Classics in Chemical Neuroscience: Cocaine. ACS Chem. Neurosci..

[37] Illés A., Balicza P., Molnár V., Bencsik R., Szilvási I., Molnar M.J. (2019). Dynamic interaction of genetic risk factors and cocaine abuse in the background of Parkinsonism—A case report. BMC Neurol..

[38] Vaillancourt D.E., Bschonfeld D., Kwak Y., Bohnen N.I., Seidler R. (2013). Dopamine overdose hypothesis: Evidence and clinical implications. Mov. Disord..

[39] Cadet J.L., Brannock C. (1998). Free radicals and the pathobiology of brain dopamine systems. Neurochem. Int..

[40] Granado N., Ares-Santos S., Moratalla R. (2013). Methamphetamine and Parkinson’s Disease. Park. Dis..

[41] Granado N., Ares-Santos S., O’Shea E., Vicario-Abejón C., Colado M.I., Moratalla R. (2010). Selective Vulnerability in Striosomes and in the Nigrostriatal Dopaminergic Pathway After Methamphetamine Administration: Early loss of TH in striosomes after methamphetamine. Neurotox. Res..

[42] Granado N., O’Shea E., Bove J., Vila M., Colado M.I., Moratalla R. (2008). Persistent MDMA-induced dopaminergic neurotoxicity in the striatum and substantia nigra of mice. J. Neurochem..

[43] Yamamoto B.K., Zhu W. (1998). The effects of methamphetamine on the production of free radicals and oxidative stress. J. Pharmacol. Exp. Ther..

[44] Büttner A. (2011). Review: The neuropathology of drug abuse. Neuropathol. Appl. Neurobiol..

[45] Bernstein A.I., Stout K.A., Miller G.W. (2014). The vesicular monoamine transporter 2: An underexplored pharmacological target. Neurochem. Int..

[46] Little K.Y., Ramssen E., Welchko R., Volberg V., Roland C.J., Cassin B. (2009). Decreased brain dopamine cell numbers in human cocaine users. Psychiatry Res..

[47] Villalba R.M., Smith Y. (2013). Differential striatal spine pathology in Parkinson’s disease and cocaine addiction: A key role of dopamine?. Neuroscience.

[48] Dumitriu D., LaPlant Q., Grossman Y.S., Dias C., Janssen W.G., Russo S.J., Morrison J.H., Nestler E.J. (2012). Subregional, Dendritic Compartment, and Spine Subtype Specificity in Cocaine Regulation of Dendritic Spines in the Nucleus Accumbens. J. Neurosci..

[49] Dobi A., Seabold G.K., Christensen C.H., Bock R., Alvarez V.A. (2011). Cocaine-Induced Plasticity in the Nucleus Accumbens Is Cell Specific and Develops without Prolonged Withdrawal. J. Neurosci..

[50] Kolb B., Gorny G., Li Y., Samaha A.-N., Robinson T.E. (2003). Amphetamine or cocaine limits the ability of later experience to promote structural plasticity in the neocortex and nucleus accumbens. Proc. Natl. Acad. Sci. USA.

[51] Pulipparacharuvil S., Renthal W., Hale C.F., Taniguchi M., Xiao G., Kumar A., Russo S.J., Sikder D., Dewey C.M., Davis M.M. (2008). Cocaine Regulates MEF2 to Control Synaptic and Behavioral Plasticity. Neuron.

[52] Li J., Liu N., Lu K., Zhang L., Gu J., Guo F., An S., Zhang L., Zhang L. (2012). Cocaine-induced dendritic remodeling occurs in both D1 and D2 dopamine receptor-expressing neurons in the nucleus accumbens. Neurosci. Lett..

[53] Lee K.-W., Kim Y., Kim A.M., Helmin K., Nairn A.C., Greengard P. (2006). Cocaine-induced dendritic spine formation in D1 and D2 dopamine receptor-containing medium spiny neurons in nucleus accumbens. Proc. Natl. Acad. Sci. USA.

[54] Villalba R.M., Smith Y. (2010). Striatal Spine Plasticity in Parkinson’s Disease. Front. Neuroanat..

[55] Tian X., Kai L., Hockberger P.E., Wokosin D.L., Surmeier D.J. (2010). MEF-2 regulates activity-dependent spine loss in striatopallidal medium spiny neurons. Mol. Cell. Neurosci..

[56] Clare K., Pan C., Kim G., Park K., Zhao J., Volkow N.D., Lin Z., Du C. (2021). Cocaine Reduces the Neuronal Population While Upregulating Dopamine D2-Receptor-Expressing Neurons in Brain Reward Regions: Sex-Effects. Front. Pharmacol..

[57] Friedman J.H., Chang V. (2013). Crack cocaine use due to dopamine agonist therapy in Parkinson disease. Neurology.

[58] Mash D.C., Ouyang Q., Pablo J., Basile M., Izenwasser S., Lieberman A., Perrin R.J. (2003). Cocaine Abusers Have an Overexpression of α-Synuclein in Dopamine Neurons. J. Neurosci..

[59] Kon T., Tomiyama M., Wakabayashi K. (2020). Neuropathology of Lewy body disease: Clinicopathological crosstalk between typical and atypical cases. Neuropathology.

[60] Todd G., Noyes C., Flavel S.C., Della Vedova C.B., Spyropoulos P., Chatterton B., Berg D., White J.M. (2013). Illicit Stimulant Use Is Associated with Abnormal Substantia Nigra Morphology in Humans. PLoS ONE.

[61] Siniscalchi A., Bonci A., Mercuri N.B., De Siena A., De Sarro G., Malferrari G., Diana M., Gallelli L. (2015). Cocaine Dependence and Stroke: Pathogenesis and Management. Curr. Neurovascular Res..

[62] Tamrazi B., Almast J. (2012). Your Brain on Drugs: Imaging of Drug-related Changes in the Central Nervous System. RadioGraphics.

[63] Serafini G., Piccinini G., Visimberga S., Cervetti A., Belvederi Murri M., Monacelli F., Pompili M., Amore M. (2019). Aripiprazole-Induced Persistent Hiccup: A Case Report and Review of the Literature. Psychiatr. Danub..

[64] Carbone M.G., Tagliarini C., Della Rocca F., Flamini W., Pagni G., Tripodi B., Marazziti D., Maremmani I. (2021). Protracted Hiccups Induced by Aripiprazole and Regressed after Administration of Gabapentin. Case Rep. Psychiatry.

[65] Loonen A.J., Ivanova S.A. (2021). Neurobiological mechanisms associated with antipsychotic drug-induced dystonia. J. Psychopharmacol..

[66] APA (2013). Diagnostic and Statistical Manual of Mental Disorders: DSM-5.

